# A dataset for climate, land, energy and water systems modelling in Lao PDR

**DOI:** 10.1016/j.dib.2025.112084

**Published:** 2025-09-19

**Authors:** Annie Flint Smith, Kane Alexander, Fernando A. Plazas-Niño, Mark Howells

**Affiliations:** a Centre for Environmental Policy, Imperial College London, London, SW7 2BX, UK; b STEER Centre, Department of Geography, Loughborough University, Loughborough, LE11 3TU, UK

**Keywords:** Resource modelling, CLEWs, OSeMOSYS, Power generation, Land cover, Emissions, Water Use

## Abstract

Policymaking often treats economic and resource sectors in isolation of one another, neglecting to consider the interconnections between a country’s climate, land, energy and water (CLEW) systems. This approach can lead to system inefficiencies and unintended consequences, as the synergies and trade-offs between sectors are overlooked. A nexus-thinking approach addresses this issue by considering multiple resources and their interlinkages in the analytical and decision-making processes, enabling policymakers to make more informed decisions. This article presents a dataset containing techno-economic data inputs specific to the Lao PDR’s (Laos’) CLEW systems, in the context that Laos’ policymaking has traditionally treated sectors independently. The CLEWs systems data were collected from various open-access resources, including online repositories and national reports, with the objective to facilitate integrated resource system modelling assessments for Laos that incorporate a nexus-thinking approach. To support this objective, this article also describes the calculations used to process raw data to meet typical systems modelling software requirements, using the Open-Source Energy Modelling Systems (OSeMOSYS) framework as a reference. By including greenhouse gas emission factors, this dataset provides the basis for long-term scenario planning that can inform Laos’ national decarbonisation strategy, whilst accounting for other potential cross-sector dynamics. The structure and documentation of this dataset also has significant reuse potential, as it lays the foundation for other researchers to replicate similar CLEW datasets for resource system assessments in other countries with similar economic and environmental backgrounds.

Specifications Table

**Table:** 

Subject	Renewable Energy, Sustainability, and the Environment
Specific subject area	Climate, land, energy and water systems modelling
Type of data	Table Raw, Analysed, Processed
Data collection	All data were collected from public sources, including dataset repositories, websites, and online reports published by national entities. Where country-specific data were not available, global or regional data were collected.
Data source location	The raw data sources are detailed in their respective sections throughout this article.
Data accessibility	Repository name: Zenodo Data identification number: https://doi.org/10.5281/zenodo.14282913 Direct URL to data: https://zenodo.org/records/14282913

## 1 Value of the Data

• This dataset is valuable for conducting energy-water-food nexus studies, as it collates national climate, land, energy, and water data into a single, more easily accessible resource, helping to identify synergies and trade-offs that have often been overlooked in academic research and policy decision-making.
• This dataset provides key stakeholders in Laos, particularly policymakers, to evaluate the wider sectoral and resource system consequences of implementing specific greenhouse gas mitigation measures.
• This dataset can be reused by other researchers to build a more comprehensive climate, land, energy and water systems model for Laos, for further policy scenario assessments, particularly with regards to Laos’ national decarbonisation strategy.
• The structure of this dataset is designed to be interoperable with different open-source modelling tools to promote reusability. By using standard data tables and file formats (e.g. CSV, XLSX), the dataset facilitates easy integration with different system machines and resource toolsIn particular, the dataset is designed to integrate efficiently with the Open-Source Energy Modelling System (OSeMOSYS), a tool widely used for least-cost energy system optimisation, scenario analysis, and long-term decarbonisation planning. It can also be adapted for use in tools such as the Long-range Energy Alternatives Planning (LEAP) tool and the Integrated MARKAL-EFOM System (TIMES). By integrating with these tools, the dataset supports the resolution of core problems in energy-water-food systems planning, including quantifying cross-sectoral trade-offs, assessing the consequences of resource constraints, and identifying least-cost pathways within the context of decarbonisation efforts and socio-economic targets.
• Other researchers can also use this dataset as a basis for modelling resource system interlinkages and assessing future decarbonisation policy scenarios in similar countries.

## 2 Background

As one of the 195 signatories to the Paris Agreement, Laos is obligated to submit its latest Nationally Determined Contribution (NDC) by the end of 2025 [1]. This requires a holistic, evidence-based approach to decision-making that considers the synergies and trade-offs across interconnected resource systems, including climate, land, energy, and water (CLEW). This dataset compiles Laos’ CLEW data to address the frequent omission of resource system interlinkages in academic research and policymaking. The dataset was developed to support integrated resource systems modelling using open-source tools such as the Open-Source Energy Modelling System (OSeMOSYS). Whilst previous research efforts have produced national-level technoeconomic datasets to support resource system decision-making, these have predominantly been sector-specific, with a focus on the energy sector [2,3]. By compiling Laos’ CLEW data into an open-access resource, this dataset facilitates and promotes nexus-focused research for future policymaking in Laos and elsewhere.

## 3 Data Description

The dataset presented in this article contains key techno-economic data inputs specific to Laos, which can be used to create an integrated climate, land, energy, and water systems (CLEWs) model. Whilst the dataset can be used with any resource system modelling tool, it was developed with reference to the Open-Source Energy Modelling System (OSeMOSYS) tool. OSeMOSYS was chosen as a reference point for processing and formatting data inputs because it has been previously used to develop sector-specific datasets, such as the Laos’ Energy Starter Data Kit (SDK) [4]. Nonetheless, the dataset is tool-agnostic and can be adapted for use in a range of resource modelling software that can utilise csv and excel file formats. Users should, however, ensure that the additional socio-economic parameters of alternative tools are met; for example, the Long-range Energy Alternatives Planning (LEAP) system requires additional data inputs, such as rates of urbanisation and average household sizes [5].

Following the methodology outlined in Laos’ Energy SDK collection process [4], the data were sourced exclusively from publicly accessible online resources, including national reports and international databases. The Food and Agriculture Organisation (FAO) database was utilised for land and water data inputs, with the latter primarily sourced through the FAO’s AQUASTAT dataset. Laos’ SDK [4] outlined the techno-economic data inputs that should be considered for each sector and also served as a foundational resource for energy-specific data inputs. Data were cross validated by comparing figures across public datasets, and where there were discrepancies, the most recent or nationally endorsed data were prioritised. It should be noted for future users that data availability varied by sector. For instance, the SDK compiled by Climate Compatible Growth [4] provided more readily available techno-economic inputs for the energy sector compared to land or water. Where there were knowledge gaps, proxy data were used and typically standardised to remain the same throughout the modelling period, due to a lack of information on the socio-economic drivers that may affect techno-economic parameters in the long-term. Section 4 of this paper outlines the data sources used to produce this dataset, as well as the calculative methods required to process data where necessary.

To improve future replicability for researchers aiming to build or extend specific sectors, the dataset in this section is organised by resource system and grouped into three categories: energy, land, and water. The complete dataset is available in the Zenodo repository, under the title ‘Techno-economic climate, land, energy and water systems dataset for long-term decarbonisation modelling in Laos’ [6]. The DOI is provided in the specifications table in this article.

*Index*

**Table:** 

Item	Main Data Source(s)	Description of Contents
Table 1	Public datasets (international database, research initiative)	Historic power generation in Laos (2020–2023)
Table 2	Public datasets (international database)	Laos’ GDP (2019–2023)
Table 3	Public datasets (international database)	Electricity consumption per sector (2020–2023)
Table 4	Public datasets (international databases)	Projected electricity consumption per sector, based on IEA data and GDP growth rate
Table 5	Public dataset (research initiative)	Capital costs of generation technologies (2020–2055)
Table 6	Public dataset (research initiative)	Capital costs of transmission and distribution technologies (2020–2055)
Table 7	Public dataset (research initiative)	Fixed costs of generation technologies (2020–2055)
Table 8	Public dataset (research initiative)	Fixed costs of transmission and distribution technologies (2020–2055)
Table 9	Public dataset (research initiative)	Emissions factors for fuel-based technologies
Table 10	Public dataset (research initiative)	Operational life of generation technologies (2020–2055)
Table 11	Public dataset (research initiative)	Operational life of transmission and distribution technologies (2020–2055)
Table 12	Public dataset (research initiative)	Estimated average efficiencies for generation technologies
Table 13	Public dataset (research initiative)	Estimated average efficiencies for transmission and distribution technologies
Table 14	Public dataset (research initiative)	Estimated average capacity factors for generation technologies
Table 15	Public dataset (research initiative)	Estimated average capacity factors for transmission and distribution technologies
Table 16	Public datasets (international database and research initiative)	Residual capacities for generation technologies (2020–2055)
Table 17	Public datasets (international database and research initiative)	Residual capacities for transmission and distribution technologies (2020–2055)
Table 18	Public datasets (international databases)	Geophysical parameters in Laos
Table 19	International organisation report	Laos’ Population
Table 20	Public datasets (international databases)	Crop production and trade data
Table 21	Public datasets (international databases)	Crop demand in Laos
Table 22	Public datasets (international databases)	Minimum projected land cover in Laos (2020–2055)
Table 23	Public dataset (research initiative)	Capital costs of land technologies (2020–2055)
Table 24	Public market data aggregator	Variable costs of land technologies (2020–2055)
Table 25	Public dataset (research initiative)	Operational lifetime of Laos’ main crops
Table 26	Public datasets (international organisation, research paper)	Output activity ratios of Laos’ main crops
Table 27	Public dataset (research initiative)	Residual capacities of Laos’ main crops
Table 28	Public dataset; Research literature	Public water demand
Table 29	Public dataset (research initiative)	Water-specific input activity ratios for all energy technologies
Table 30	Public datasets (international databases)	Water-specific input activity ratios for all land technologies
Table 31	Public dataset	Output activity ratios for crop technologies and water commodities
Table 32	Public dataset	Output activity ratios for land cover technologies and water commodities
Table 33	N/A	List of sources for Laos’ historic power generation
Table 34	N/A	List of sources for Laos’ GDP data
Table 35	N/A	List of sources for electricity consumption per sector
Table 36	N/A	List of sources for the residual capacities of energy-specific technologies
Table 37	N/A	List of sources for Laos’ geophysical parameters
Table 38	N/A	List of sources for Laos’ crop production and trade data
Table 39	N/A	List of sources for the output activity ratios for land technologies
Table 40	N/A	List of sources for water-specific input activity ratios

### 3.1 Energy

#### 3.1.1 Demands

Laos’ future electricity demand up to 2070 was projected based on the country’s historic electricity consumption (see Table 3) and the current GDP growth rate (see Table 2). Table 1 shows how Laos' previous electricity demand has been met through coal, hydro, biofuel and solar power generation. As shown in Table 3, Laos’ electricity demand is segregated into three sectors: industrial, commercial, and residential. The calculations used for projecting the sector-specific electricity demand are presented in Section 4.1.1. and the complete dataset is available in the ‘Laos Model Configuration & Inputs’ excel file in the Zenodo repository, under the tab “Demands”. Table 4 presents the projected electricity demand on a 10-year basis.

**Table 1:** Historic power generation in Laos (2020–2022).

Technology	Electricity Generation (PJ)		
	2020	2021	2022
Coal Power Plant	40.878	42.772	43.844
Hydro Power Plant	102.65	118.50	139.45
Biofuels	0.21	0.17	0.13
Solar PV	0.15	0.24	0.28
**Total**	**143.88**	**161.69**	**183.71**

**Table 2:** Laos’ GDP (2019–2023).

Parameter	Unit	Year				
		2019	2020	2021	2022	2023
GDP	Constant 2015 US$ Billion	18.49	19.59	19.05	19.57	20.3
GDP Growth Rate	%	5.5	0.5	2.5	2.7	3.7

**Table 3:** Electricity consumption per sector (2020–2022).

Sector	Annual Demand (PJ)		
	2020	2021	2022
Industrial	8.812	9.079	9.346
Commercial	4.436	7.987	8.222
Residential	7.752	4.57	4.705

**Table 4:** Projected electricity demand per sector, based on IEA data and GDP growth rate.

Sector	Projected Annual Demand (PJ)					
	2020	2030	2040	2050	2060	2070
Industrial	8.812	11.588	14.563	16.767	18.734	20.931
Commercial	4.436	5.834	7.332	8.441	9.432	10.538
Residential	7.752	10.194	12.811	14.75	16.48	18.414
Industrial	8.812	11.588	14.563	16.767	18.734	20.931
**Total**	**21.000**	**27.616**	**34.706**	**39.958**	**44.646**	**49.883**

#### 3.1.2 Capital costs

In the energy sector, capital costs represent the initial investment needed to build or acquire a generation technology. Table 5, Table 6 show the capital costs every 10 years for Laos’ different energy technologies. The cost values of all fossil fuel power plants are kept stable throughout the modelling period, ranging from $1000 million per petajoule (/PJ) to $2750 million/PJ. This is due to the absence of available information on how these costs may change in future years. This approach aligns with established practice in long-term modelling where forward-looking data is unavailable [7]. The only exceptions are the onshore wind and solar PV technologies, which are assumed to decrease in the first twenty years. This assumption is based on values provided in the data source, the Starter Data Kit composed by Climate Compatible Growth [4]. It is, however, improbable that capital costs would remain the same over extended periods; readers are therefore recommended to consider market dynamics and proposed policy interventions when adapting these cost values for future analyses.

**Table 5:** Capital costs of generation technologies (2020–2070).

Technology	Capital Cost (Million USD$/PJ)					
	2020	2030	2040	2050	2060	2070
Coal power plant	1300	1300	1300	1300	1300	1300
Large Hydro (>100MW)	1539	1539	1539	1539	1539	1539
Onshore wind	2220	1621	1391	1391	1391	1391
Solar PV (Utility)	1160	746	609	609	609	609
Biomass power plant	2750	2750	2750	2750	2750	2750
Geothermal Power Plant	2500	2500	2500	2500	2500	2500
Gas power plant (CCGT)	1000	1000	1000	1000	1000	1000
Light fuel oil power plant	1200	1200	1200	1200	1200	1200
Oil fired gas turbine (SCGT)	1344	1344	1344	1344	1344	1344
Light Fuel Oil Standalone Generator (1 kW)	1500	1500	1500	1500	1500	1500

**Table 6:** Capital costs of transmission and distribution technologies (2020–2070).

Technology	Capital Cost (Million USD$/PJ)					
	2020	2030	2040	2050	2060	2070
Electricity transmission	204.27	204.27	204.27	204.27	204.27	204.27
Electricity distribution	102.13	102.13	102.13	102.13	102.13	102.13

The capital costs on an annual basis are available under the tab ‘Capital Costs’ in the aforementioned excel file on Zenodo.

#### 3.1.3 Fixed costs

Fixed costs refer to annual operation and maintenance costs that are associated with a specific technology, which do not vary with the level of production [8]. These may vary by technology but in the power sector, fixed costs typically include staff salaries, routine machinery maintenance, insurance, and administrative overheads required to keep the plant operational.

Excluding solar PVs and onshore wind, the fixed costs of Laos’ generation technologies remain constant in multiple study years, ranging from $18 million/PJ to $100 million/PJ, depending on the fuel source. This is shown in Table 7. Table 8 displays the fixed costs of Laos’ transmission and distribution technologies, which are assumed to stay at $4.09 million/PJ and $2.04 million/PJ respectively.

**Table 7:** Fixed costs of generation technologies (2020–2070).

Technology	Fixed Cost (Million USD$/PJ)					
	2020	2030	2040	2050	2060	2070
Coal power plant	52.00	52.00	52.00	52.00	52.00	52.00
Large Hydro (>100MW)	46.17	46.17	46.17	46.17	46.17	46.17
Onshore wind	88.80	64.83	55.64	55.64	55.64	55.64
Solar PV (Utility)	15.08	9.70	7.91	7.91	7.91	7.91
Biomass power plant	69.00	69.00	69.00	69.00	69.00	69.00
Geothermal Power Plant	100.00	100.00	100.00	100.00	100.00	100.00
Gas power plant (CCGT)	40.00	40.00	40.00	40.00	40.00	40.00
Light fuel oil power plant	18.00	18.00	18.00	18.00	18.00	18.00
Oil fired gas turbine (SCGT)	18.00	18.00	18.00	18.00	18.00	18.00
Light Fuel Oil Standalone Generator (1 kW)	38.00	38.00	38.00	38.00	38.00	38.00

**Table 8:** Fixed costs of transmission and distribution technologies (2020–2070).

Technology	Fixed Cost (Million USD$/PJ)					
	2020	2030	2040	2050	2060	2070
Electricity transmission	4.09	4.09	4.09	4.09	4.09	4.09
Electricity distribution	2.04	2.04	2.04	2.04	2.04	2.04

This is a common assumption in long-term systems modelling to assume the values remain constant [7] and this practice is adopted here due to limited data on how these costs may evolve over time. It is recognised that in practice, fixed costs may change over time due to factors such as inflation or technological developments, but projecting such changes over a 50-year time period would introduce significant uncertainty to the dataset and future modelling. Therefore, maintaining constant values across the modelling period helps to ensure transparency and reproducibility, and provides flexibility for future users to modify this assumption should more context-specific data become available. As shown in Table 7, the fixed costs of Laos’ onshore wind and solar PV technologies vary between 2020–2040, due to additional context-specific information available in the original SDK resource [4].

The complete data is available in the excel file under the ‘Fixed Cost’ tab.

#### 3.1.4 Emission factors

In the energy sector, the emission factor represents the amount of carbon dioxide released per unit of energy produced using a specific fuel. Table 9 shows the CO_2_ emission factor for each fuel used in Laos’ energy sector, in kilograms per gigajoule. These values do not vary over time; for instance, the emission factor for coal is 94.6 kgCO2/GJ and for natural gas is 56.1 kgCO2/GJ across all study years.

**Table 9:** Emissions factors for fuel-based technologies.

Fuel	CO_2_ Emission Factor (kg/GJ)
Crude Oil	73.3
Biomass	100
Coal	94.6
Natural Gas	56.1
Diesel for Agriculture	69.8

This data is also available under the tab ‘Emission Factor’ in the same excel file as previously mentioned.

#### 3.1.5 Operational lifetimes

The operational lifetime of a technology refers to the duration it is expected to function efficiently and reliably after its deployment. Table 10 shows the operational lifetimes of each generation technology in Laos. Similarly, Table 11 shows the operational lifetimes of Laos’ electricity transmission and distribution technologies. This data is also available under the tab ‘Operational Lifetime’ in the excel file.

**Table 10:** Operational life of generation technologies (2020–2070).

Technology	Operational Life (Years)
Coal power plant	60
Large Hydro (>100MW)	40
Onshore wind	30
Solar PV (Utility)	30
Biomass power plant	25
Geothermal Power Plant	50
Gas power plant (CCGT)	30
Light fuel oil power plant	50
Oil fired gas turbine (SCGT)	50
Light Fuel Oil Standalone Generator (1 kW)	20

**Table 11:** Operational life of transmission and distribution technologies (2020–2070).

Technology	Operational Life (Years)
Electricity transmission	50
Electricity distribution	70

#### 3.1.6 Efficiencies

The efficiency of an energy sector technology is measured by comparing the energy input from its fuel source to the energy it produces in any given year, or across a chosen period. Table 12 presents the estimated average efficiencies for Laos’ energy generation technologies. Table 13 shows the estimated average efficiencies for Laos’ transmission and distribution technologies.

**Table 12:** Estimated average efficiencies for generation technologies.

Technology	Efficiency (%)
Coal power plant	30
Large Hydro (>100MW)	100
Onshore wind	100
Solar PV (Utility)	100
Biomass power plant	38
Geothermal Power Plant	10
Gas power plant (CCGT)	55
Light fuel oil power plant	40
Oil fired gas turbine (SCGT)	40
Light Fuel Oil Standalone Generator (1 kW)	42

**Table 13:** Estimated average efficiencies for transmission and distribution technologies.

Technology	Efficiency (%)
Electricity transmission	100
Electricity distribution	100

The data used to calculate the energy efficiencies can be found in the ‘Laos Model Configuration & Inputs’ excel file; the inputs are listed under the ‘Input Activity Ratio’ tab, and the outputs are listed under the ‘Output Activity Ratio’ tab.

#### 3.1.7 Capacity factors

The capacity factor measures how efficiently a technology is utilised, by comparing the actual energy produced over a specific period to the maximum possible output it could achieve if operating at full capacity the entire time. Table 14, Table 15 present the average capacity factor for each of Laos’ generation, transmission and distribution technologies. However, the complete data is available in the excel file under the ‘Capacity Factor’ tab. In this dataset, capacity factors are calculated across 8 different time slices to reflect variations in energy generation throughout the day. For example, solar PVS cannot generate electricity at night, resulting in a capacity factor of 0 during these time slices.

**Table 14:** Estimated average capacity factors for generation technologies.

Technology	Average Capacity Factor (%)
Coal power plant	75
Large Hydro (>100MW)	55
Onshore wind	8
Solar PV (Utility)	14
Biomass power plant	70
Geothermal Power Plant	70
Gas power plant (CCGT)	55
Light fuel oil power plant	25
Oil fired gas turbine (SCGT)	25
Light Fuel Oil Standalone Generator (1 kW)	40

**Table 15:** Estimated average capacity factors for transmission and distribution technologies.

Technology	Average Capacity Factor (%)
Electricity transmission	100
Electricity distribution	100

#### 3.1.8 Residual capacities

Residual capacity in the energy sector represents the remaining installed capacity of any power generation technology. Assuming usage and operations remain stable, Laos’ on-grid residual capacity data is presented in Table 16 on a 10-year basis. Table 17 shows the residual capacity for Laos’ electricity transmission and distribution networks. This data appears constant; this is because the operational lifetime of these technologies extends beyond the total modelling period. This data is also available on an annual basis in the excel file under the tab ‘Residual Capacity’.

**Table 16:** Residual capacities for generation technologies (2020–2070).

Technology	Residual Capacity (GW)					
	2020	2030	2040	2050	2060	2070
Coal power plant	1.878	6.338	6.338	4.46	4.46	4.46
Large Hydro (>100MW)	7.208	11.714	13.252	13.176	8.048	3.59
Onshore wind	0	0.6	0.6	0.6	0	0
Solar PV (Utility)	0	0.064	0.064	0.064	0	0
Biomass power plant	0.03	0.03	0	0	0	0
Geothermal Power Plant	0	0	0	0	0	0
Gas power plant (CCGT)	0	0	0	0	0	0
Light fuel oil power plant	0	0	0	0	0	0
Oil fired gas turbine (SCGT)	0	0	0	0	0	0
Light Fuel Oil Standalone Generator (1 kW)	0	0	0	0	0	0

**Table 17:** Residual capacities for transmission and distribution technologies (2020–2070).

Technology	Residual Capacity (GW)					
	2020	2030	2040	2050	2060	2070
Electricity transmission	6.36	6.36	6.36	6.36	6.36	6.36
Electricity distribution	6.36	6.36	6.36	6.36	6.36	6.36

### 3.2 Land

#### 3.2.1 Demands

Laos’ land data is classified into five distinct land types: forest, water bodies, built-up areas, agricultural land, and other land. The last land type refers to all grassland, wetland, shrubland, sparse vegetation, and bare area in the country. Table 18 outlines the land cover in Laos for each of these land types from 2000 to 2020, and Table 22 depicts the projected land cover up to 2070. Laos’ water bodies and ‘other land’ area is assumed to remain unchanged across the modelling period, with a total land cover of 2.256 10^3^km^2^ and 55.56 10^3^km^2^, respectively. This data is presented on an annual basis under the ‘Demands’ tab in the ‘Laos Model Configuration & Inputs’ excel file on Zenodo.

**Table 18:** Geophysical parameters in Laos.

Parameter	Land Cover (10^3^km^2^)				
	2000	2005	2010	2015	2020
Country Size	236.80	236.80	236.80	236.80	236.80
Forest (Tree) Cover	132.65	130.96	130.02	130.30	130.26
Built-up land	0.05	0.08	0.10	0.16	0.16
Water Bodies	1.98	1.99	2.07	2.14	2.21
Agriculture land	39.77	41.334	42.44	42.59	42.98
Other land	62.35	62.436	62.17	61.61	61.19
Irrigation Potential	-	-	-	-	6.00
Cultivated area equipped for irrigation (expressed as %)	-	-	-	-	32.57

**Table 19:** Laos’ Population (2019–2023).

Parameter	Unit	Year				
		2019	2020	2021	2022	2023
Population	Million	7.2	7.3	7.5	7.6	7.7
Population Growth Rate	%	1.54	1.5	1.53	1.5	1.46

Laos’ agriculture sector is also represented within the land data and plays a crucial role in determining future land use within the CLEWs model. Table 20 outlines the production, planted areas and trade data for Laos’ main crops. These crops were identified based on their large harvests and production quantities in comparison to the rest of Laos’ agriculture sector. The seven chosen crops account for 91.6 % of Laos’ total harvested area and 93.1 % of the total crop production. Table 21 outlines the projected demand on a 10-year basis for each of these crops; crop demand was projected using Laos' population growth rate as a driver (Table 19). This data is also available on an annual basis under the ‘Demands’ tab in the excel file.

**Table 20:** Crop production and trade data for 2022.

Technology	Crop production and trade data for 2022			
	Area Harvested (10^3^km^2^)	Production Quantity (mTon)	Import Quantity (mTon)	Export Quantity (mTon)
Rice	8.18	3.6	0	0.008
Cassava	1.95	5.28	0.009	1.95
Fresh vegetables	1.76	1.5	0	0.00001
Sugarcane	0.30	1.5	0.005	1.099
Maize	1.31	0.68	0.004	0.069
Banana	0.31	0.93	0.0001	0.077
Coffee	0.83	0.16	0.0005	0.204

**Table 21:** Crop demand in Laos.

Crop	Annual demand (mTon)					
	2020	2030	2040	2050	2060	2070
Rice	3.567	4.079	4.664	5.334	6.099	6.974
Cassava	1.162	1.329	1.519	1.737	1.987	2.272
Fresh vegetables	1.858	2.125	2.43	2.778	3.177	3.633
Sugarcane	1.489	1.703	1.947	2.226	2.546	2.911
Maize	0.492	0.563	0.643	0.736	0.841	0.962
Banana	0.731	0.836	0.956	1.093	1.25	1.429
Coffee	0.121	0.138	0.158	0.181	0.207	0.237

**Table 22:** Minimum projected land cover in Laos (2020–2070).

Land Type	Land Cover (10^3^km^2^)					
	2020	2030	2040	2050	2060	2070
Forests	129.6	165.76	165.76	165.76	165.76	165.76
Built-up areas	0.095	0.134	0.19	0.288	0.393	0.537
Water Bodies	2.256	2.256	2.256	2.256	2.256	2.256
Other land	55.56	55.56	55.56	55.56	55.56	55.56

#### 3.2.2 Crop capital costs

Table 23 presents the capital costs for Laos’ crop technologies on a 10-year basis. Capital costs refer to initial annual investments assumed to be available at the beginning of each calendar year [7]. In the agriculture sector, capital costs include the purchase of necessary farming machinery, such as tractors, harvesters, and irrigation systems. Besides machinery, capital costs also can include the initial purchasing of farmland and buildings, and initial set up costs, such as land clearing and facilities construction. These costs are assumed constant across the modelling horizon; rainfed crops remain at $10 million per 10^3^km^2^ and irrigated crops remain at $80 million per 10^3^km^2^. This data is also available in the excel file under the ‘Capital Cost’ tab.

**Table 23:** Capital costs of land technologies (2020–2070).

Crop	Capital Cost (Million USD$/10^3^km^2^)					
	2020	2030	2040	2050	2060	2070
Rainfed rice	10	10	10	10	10	10
Irrigated rice	80	80	80	80	80	80
Rainfed cassava	10	10	10	10	10	10
Irrigated cassava	80	80	80	80	80	80
Rainfed fresh vegetables	10	10	10	10	10	10
Irrigated fresh vegetables	80	80	80	80	80	80
Rainfed sugarcane	10	10	10	10	10	10
Irrigated sugarcane	80	80	80	80	80	80
Rainfed maize	10	10	10	10	10	10
Irrigated maize	80	80	80	80	80	80
Rainfed banana	10	10	10	10	10	10
Irrigated banana	80	80	80	80	80	80
Rainfed coffee	10	10	10	10	10	10
Irrigated coffee	80	80	80	80	80	80

#### 3.2.3 Variable costs

This study utilises the variable cost parameter for land technologies to influence future land distribution. The specific variable costs assigned to each land type are detailed in Table 24. As Laos’ forest cover has steadily declined over the last 20 years (see Table 18), it was deemed unrealistic that this land type should be given a negative variable cost as this would result in the model allocating future land to forests. Therefore, the ‘Other land’ technology was given a negative variable cost, to represent the uncertainty around how Laos’ land cover will change up to 2070.

**Table 24:** Variable costs of land technologies (2020–2070).

Land Technology	Variable Cost (Million USD$/10^3^km^2^)					
	2020	2030	2040	2050	2060	2070
Forests	0	0	0	0	0	0
Built-up areas	2	2	2	2	2	2
Water bodies	2	2	2	2	2	2
Other land	−2	−2	−2	−2	−2	−2
Rice imports	1.16	1.16	1.16	1.16	1.16	1.16
Cassava imports	0.61	0.61	0.61	0.61	0.61	0.61
Fresh vegetable imports	0.86	0.86	0.86	0.86	0.86	0.86
Sugarcane imports	1.46	1.46	1.46	1.46	1.46	1.46
Maize imports	2.44	2.44	2.44	2.44	2.44	2.44
Banana imports	1.21	1.21	1.21	1.21	1.21	1.21
Coffee imports	2.4	2.4	2.4	2.4	2.4	2.4
Rainfed rice	0.0001	0.0001	0.0001	0.0001	0.0001	0.0001
Irrigated rice	0.0001	0.0001	0.0001	0.0001	0.0001	0.0001
Rainfed cassava	0.0001	0.0001	0.0001	0.0001	0.0001	0.0001
Irrigated cassava	0.0001	0.0001	0.0001	0.0001	0.0001	0.0001
Rainfed fresh vegetables	0.0001	0.0001	0.0001	0.0001	0.0001	0.0001
Irrigated fresh vegetables	0.0001	0.0001	0.0001	0.0001	0.0001	0.0001
Rainfed sugarcane	0.0001	0.0001	0.0001	0.0001	0.0001	0.0001
Irrigated sugarcane	0.0001	0.0001	0.0001	0.0001	0.0001	0.0001
Rainfed maize	0.0001	0.0001	0.0001	0.0001	0.0001	0.0001
Irrigated maize	0.0001	0.0001	0.0001	0.0001	0.0001	0.0001
Rainfed banana	0.0001	0.0001	0.0001	0.0001	0.0001	0.0001
Irrigated banana	0.0001	0.0001	0.0001	0.0001	0.0001	0.0001
Rainfed coffee	0.0001	0.0001	0.0001	0.0001	0.0001	0.0001
Irrigated coffee	0.0001	0.0001	0.0001	0.0001	0.0001	0.0001

Modellers may wish to use a default high variable cost value (i.e. $9999) for crop import technologies to defer the model from choosing to import crops rather than produce domestically. However, Table 24 provides the estimated variable cost of each crop import based on available data. This value was kept constant across the modelling period due to a lack of available data on how crop import prices may change in the future. For example, the variable cost of rice imports is held constant at $1.16 million between 2020 and 2070, while maize imports remain at $2.44 million for the same period. This data is also available under the ‘Variable Cost’ tab in the excel file.

#### 3.2.4 Operational lifetimes

Operational lifetime in the agricultural sector represents the remaining time in which a cultivated land area is still usable and productive. The number of years that cropland is suitable for is independent of the crop type, but in modelling software like OSeMOSYS, a data input for operational lifetime is required for each crop technology. Table 25 shows the operational lifetime for each of Laos’ main crops, and this data is also available in the excel file under ‘Operational Lifetime’.

**Table 25:** Operational lifetime of Laos’ main crops.

Crop Technology	Operational Life (Years)
Rainfed rice	15
Irrigated rice	15
Rainfed cassava	15
Irrigated cassava	15
Rainfed fresh vegetables	15
Irrigated fresh vegetables	15
Rainfed sugarcane	15
Irrigated sugarcane	15
Rainfed maize	15
Irrigated maize	15
Rainfed banana	15
Irrigated banana	15
Rainfed coffee	15
Irrigated coffee	15

#### 3.2.5 Crop output activity ratios

Table 26 outlines the average output activity ratio for each of Laos’ primary crops. This refers to the estimated annual yield of that crop per unit of land. A higher ratio indicates greater productivity, meaning more of that crop can be harvested from the same land area compared to crops with lower ratios.

**Table 26:** Average output activity ratios of Laos’ main crops.

Crop Technology	Average Output Activity Ratio (mTon/10^3^km^2^)
Rainfed rice	0.39
Irrigated rice	0.54
Rainfed cassava	2.41
Irrigated cassava	3.33
Rainfed fresh vegetables	0.76
Irrigated fresh vegetables	1.04
Rainfed sugarcane	4.39
Irrigated sugarcane	6.06
Rainfed maize	0.22
Irrigated maize	0.30
Rainfed banana	2.53
Irrigated banana	3.49
Rainfed coffee	0.17
Irrigated coffee	0.24

#### 3.2.6 Crop residual capacities

The residual capacity of Laos’ primary crops is presented in Table 27. This represents the cultivated area of the respective crops in a given year, taking into account the crop’s overall operational lifetime. This data can also be found under ‘Residual Capacity’ in the excel file.

**Table 27:** Residual capacities of Laos’ main crops (2020–2036).

Technology	Residual Capacity (10^3^km^2^)								
	2020	2022	2024	2026	2028	2030	2032	2034	2036
Rainfed rice	5.52	4.78	4.05	3.31	2.57	1.84	1.10	0.37	0.000
Irrigated rice	2.66	2.31	1.95	1.60	1.24	0.89	0.53	0.18	0.000
Rainfed cassava	1.31	1.14	0.96	0.79	0.61	0.44	0.26	0.09	0.000
Irrigated cassava	0.64	0.55	0.47	0.38	0.30	0.21	0.13	0.04	0.000
Rainfed fresh vegetables	1.19	1.03	0.87	0.71	0.55	0.39	0.24	0.08	0.000
Irrigated fresh vegetables	0.57	0.50	0.42	0.34	0.27	0.19	0.11	0.04	0.000
Rainfed sugarcane	0.10	0.09	0.07	0.06	0.05	0.03	0.02	0.01	0.000
Irrigated sugarcane	0.21	0.18	0.15	0.12	0.10	0.07	0.04	0.01	0.000
Rainfed maize	0.88	0.82	0.76	0.71	0.65	0.59	0.53	0.47	0.000
Irrigated maize	0.43	0.40	0.37	0.34	0.31	0.28	0.26	0.23	0.000
Rainfed banana	0.21	0.18	0.17	0.13	0.11	0.10	0.08	0.01	0.000
Irrigated banana	0.10	0.09	0.08	0.06	0.05	0.05	0.04	0.01	0.000
Rainfed coffee	0.56	0.48	0.45	0.33	0.30	0.26	0.22	0.04	0.000
Irrigated coffee	0.27	0.23	0.22	0.16	0.14	0.13	0.11	0.02	0.000

### 3.3 Water

#### 3.3.1 Demands

The demand for public water in Laos from 2020 to 2070 is presented in Table 28. For the purpose of this study, public water demand refers to the country’s total water demand, which includes both industrial and municipal water uses. This data is also available on an annual basis in the excel file under the ‘Demands’ tab.

**Table 28:** Public water demand.

Annual Demand for Public Water (10^9^m^3^)					
2020	2030	2040	2050	2060	2070
0.31	0.43	0.61	0.85	1.19	1.66

#### 3.3.2 Input activity ratios

In the energy sector, water is required to cool thermal power plants. The input activity ratio represents the amount of water needed by an energy generation technology to produce one unit of energy. Table 29 outlines the input activity ratios for Laos’ fuel-based energy generation technologies.

**Table 29:** Water-specific input activity ratios for all energy technologies.

Technology	Commodity	Input Activity Ratio (10^9^m^3^/PJ)
Coal power plant	Water for cooling in thermal power plants	0.05
Light fuel oil power plant	Water for cooling in thermal power plants	0.04
Oil fired gas turbine (SCGT)	Water for cooling in thermal power plants	0.04
Light fuel oil standalone generator (1 kW)	Water for cooling in thermal power plants	0.04

Land technologies also rely on water inputs, as shown in Table 30. In the agricultural sector, crops grown using intensive methods, such as irrigation, require additional water. The full dataset on water-specific input activity ratios is available in the excel file under ‘Input Activity Ratios.

**Table 30:** Water-specific input activity ratios for all land technologies.

Technology	Commodity	Input Activity Ratio (10^9^m^3^/10^3^km^2^)
All irrigated crops	Precipitation water	1.9
	Irrigation water	1.589
Rainfed crops	Precipitation water	1.9
	Irrigation water	0
Forests	Precipitation water	1.9
Built-up areas	Precipitation water	1.9
Water bodies	Precipitation water	1.9
Other land	Precipitation water	1.9

#### 3.3.3 Output activity ratios

Water-specific output activity ratios indicate how much water is released or lost by each land technology through processes such as evapotranspiration, groundwater recharge, and surface runoff. Table 31 presents this data for Laos’ key crops. Table 32 similarly outlines the output activity ratios for Laos’ land types. This data is also available under the ‘Output activity ratio’ tab in the excel file.

**Table 31:** Output activity ratios for crop technologies and water commodities.

Technology	Commodity	Output Activity Ratio (10^9^m^3^/10^3^km^2^)
Rainfed rice	Water lost to evapotranspiration	0.665
	Groundwater recharge	0.0627
	Surface water run-off	1.1742
Irrigated rice	Water lost to evapotranspiration	1.103
	Groundwater recharge	0.119
	Surface water run-off	2.279
Rainfed cassava	Water lost to evapotranspiration	0.368
	Groundwater recharge	0.076
	Surface water run-off	1.4554
Irrigated cassava	Water lost to evapotranspiration	0.91
	Groundwater recharge	0.13
	Surface water run-off	2.461
Rainfed fresh vegetables	Water lost to evapotranspiration	0.798
	Groundwater recharge	0.114
	Surface water run-off	0.988
Irrigated fresh vegetables	Water lost to evapotranspiration	1.47
	Groundwater recharge	0.21
	Surface water run-off	1.82
Rainfed sugarcane	Water lost to evapotranspiration	0.525
	Groundwater recharge	0.151
	Surface water run-off	2.828
Irrigated sugarcane	Water lost to evapotranspiration	1.1115
	Groundwater recharge	0.0399
	Surface water run-off	0.690
Rainfed maize	Water lost to evapotranspiration	0.57
	Groundwater recharge	0.0665
	Surface water run-off	1.2635
Irrigated maize	Water lost to evapotranspiration	1.155
	Groundwater recharge	0.119
	Surface water run-off	2.23
Rainfed banana	Water lost to evapotranspiration	0.798
	Groundwater recharge	0.114
	Surface water run-off	0.988
Irrigated banana	Water lost to evapotranspiration	1.47
	Groundwater recharge	0.21
	Surface water run-off	1.82
Rainfed coffee	Water lost to evapotranspiration	0.798
	Groundwater recharge	0.114
	Surface water run-off	0.988
Irrigated coffee	Water lost to evapotranspiration	1.47
	Groundwater recharge	0.21
	Surface water run-off	1.82

**Table 32:** Output activity ratios for land cover technologies and water commodities.

Technology	Commodity	Output Activity Ratio (10^9^m^3^/10^3^km^2^)
Forests	Water lost to evapotranspiration	1.349
	Groundwater recharge	0.0475
	Surface water run-off	0.5035
Built-up areas	Water lost to evapotranspiration	1.197
	Groundwater recharge	0.057
	Surface water run-off	0.646
Water bodies	Water lost to evapotranspiration	0.627
	Groundwater recharge	0.133
	Surface water run-off	1.14
Other land	Water lost to evapotranspiration	1.349
	Groundwater recharge	0.038
	Surface water run-off	0.513
All other crop land	Water lost to evapotranspiration	1.349
	Groundwater recharge	0.038
	Surface water run-off	0.513

## 4 Experimental Design, Materials and Methods

This section outlines the raw data sources used to put together Laos’ CLEWs techno-economic dataset. All data were compiled from free, publicly available sources, including international data repositories, national reports, and online websites. This section also provides the calculations used in the methodological process where raw data needed to be processed to meet the OSeMOSYS requirements.

### 4.1 Energy

#### 4.1.1 Demands

Table 33 lists the data sources used to determine Laos’ historic power generation in the years 2020–2023. Table 34 provides the data source used for Laos’ GDP and GDP growth rate data from 2019–2023. The annual GDP growth rate provided in the dataset was published by the World Bank Group for each year.

**Table 33:** List of sources for Laos’ historic power generation.

Energy generation technology	Sources
Coal power plant	[12]
Hydro power plant	[13]
Bioenergy	[4,14]
Solar PV	[14]

**Table 34:** List of sources for Laos’ GDP data.

Parameter	Sources
GDP	[15]
GDP Growth Rate	[15]

The sector-specific electricity consumption data was sourced from the International Energy Agency (IEA), as shown in Table 35. The end-use sectors represented – industrial, commercial, and residential – are based on the IEA’s sector classifications that underpin their national energy balances. The industrial sector aligns with the United Nation’s International Recommendations for Energy Statistics (IRES) and corresponds to ISIC Rev. 4 codes C10-C32 (manufacturing) and F41–43 (construction) [9]. Whilst the IEA does not publish detailed ISIC mappings of the residential and commercial sectors, these mirror standard international definitions; the residential sector refers to household electricity use, such as lighting, cooking and household appliances, while the commercial sector covers electricity use in service-providing facilities, like government buildings and public institutions [9]. Table 36 outlines the sources used to determine the residual capacities of energy-specific technologies.

**Table 35:** List of sources for electricity consumption per sector.

Sector	Source
Industrial sector	[16]
Commercial sector	[16]
Residential sector	[16]

**Table 36:** List of sources for the residual capacities of energy-specific technologies.

Energy technology	Sources
Coal power plant	[4,12]
Large Hydro (>100MW)	[4,13]
Onshore wind	[4]
Solar PV (Utility)	[4,14]
Biomass power plant	[4]
Geothermal Power Plant	[4]
Gas power plant (CCGT)	[4]
Light fuel oil power plant	[4]
Oil fired gas turbine (SCGT)	[4]
Light Fuel Oil Standalone Generator (1 kW)	[4]
Electricity transmission	[4]
Electricity distribution	[4]

As outlined in Eq. (1), Laos’ future electricity demand was estimated using the GDP growth rate and historic electricity consumption data for each sector. Using the GDP growth rate as a proxy driver for projecting electricity demand growth is a widely accepted method in long-tern energy planning [10]. This assumes that as the GDP growth rate increases, more electricity will be used overall; this has been observed in developing economies, where economic growth has led to higher energy use due to industrial expansion and electrification [11].

(1) Annualelectricityconsumption(PJ)=Currentconsumption×GDPgrowthrate

#### 4.1.2 Costs

All of the energy cost data used in this study was sourced from the Laos Starter Data Kit (SDK) [4]produced by Climate Compatible Growth. The Laos Starter Data Kit is a comprehensive dataset specific to Laos’ energy sector, which includes but is not limited to data on costs, residual capacities, operational lifetimes, emission factors, and input and output activity ratios, sourced from various national and international publications.

For this study’s dataset, the Laos SDK was used as the data source for all energy-specific costs, operational lifetimes, emission factors, and capacity factors.

#### 4.1.3 Operational lifetimes

The Laos SDK [1] was used as the data source for this parameter.

#### 4.1.4 Emissions factors

The Laos SDK [1] provided the emissions factors used in this study in kg/TJ, which were converted to kg/GJ for the purpose of the Laos CLEWs model. Future users should consider updating the emission factors as new technological developments and socio-economic conditions in Laos change, in order to improve the accuracy of overall emissions results in any modelling research.

#### 4.1.5 Efficiencies

Eq. (2) outlines how the efficiency of each energy generation technology was calculated. The input and output activity ratio for each technology was sourced from the Laos SDK [1].

(2) $$Efficiency\;\left(\%\right)=\frac{Output\;activity\;ratio}{Input\;activity\;ratio}$$

#### 4.1.6 Capacity factors

The Laos SDK was used to determine the capacity factor of each energy technology. As briefly explained in Section 2.1.8, the CLEWs model built for Laos included 8 time slices to represent 4 different seasons, with daytime and nighttime. There are therefore 8 capacity factors for each energy technology, which are listed in the excel file. For the purpose of this article, the capacity factors presented were calculated using a mean average of the 8 time slices for each respective technology.

#### 4.1.7 Residual capacities

The residual capacity for each energy technology was determined based on publicly available data, primarily from Global Energy Monitor. Looking at the start dates and operational lifetimes of each existing power plant, the residual capacity was calculated by summing the capacities of all plants in operation, in construction or with permits for construction.

### 4.2 Land

#### 4.2.1 Demands

The accumulated annual demand for Laos’ crop technologies was calculated by projecting the 2020 demand forward using the annual population growth rate. This assumes that as the population grows, the overall demand for food, and therefore crop production, rises proportionally.. Eq. (3) shows how annual demand is calculated using the crop production and trade data, and Eq. (4) shows how accumulated annual demand is calculated.

Table 37 lists the data sources used for Laos’ geophysical parameters and Table 38 lists the sources used for Laos’ crop production and trade data. Laos’ population data was sourced from the World Bank [17].

(3) Annualcropdemand=Productionquantity+Importquantity−Exportquantity

(4) $$Accumulated\;annual\;demand=\Sigma \left[Demand_{t}\right]$$

**Table 37:** List of sources for Botswana’s geophysical parameters.

Parameter	Sources
Country Size	[18]
Forest (Tree) Cover	[19]
Built-up land area size	[19]
Water Bodies area size	[19]
Other Land area size	[19]
Area equipped for full control irrigation	[20]
Irrigation potential	[20]
% of irrigation potential equipped for irrigation	[20]

**Table 38:** List of sources for Botswana’s crop production and trade data.

Crop	Sources			
	Area Harvested	Production	Import Quantity	Export Quantity
Rice	[21]	[21]	[22]	[22]
Cassava	[21]	[21]	[22]	[22]
Fresh vegetables	[21]	[21]	[22]	[22]
Sugarcane	[21]	[21]	[22]	[22]
Maize	[21]	[21]	[22]	[22]
Banana	[21]	[21]	[22]	[22]
Coffee	[21]	[21]	[22]	[22]

Where,

$$Demand=2020\;demand\;\times {\left(1+Population\;Growth\;Rate\right)}^{t}$$

#### 4.2.2 Costs

The capital cost data for Laos’ land technologies was sourced from Climate Compatible Growth’s resource for building a CLEWs country case study model [14]. This resource builds on the Open Learn ‘Introduction to CLEWs’ course and provides guidance on how to develop a country’s energy, land, and water systems within a model. The variable cost data for Laos’ crop import technologies was sourced from Selina Wamucii, an openly available trading exchange data web page that provides import prices for different crops for various countries [23].

#### 4.2.3 Operational lifetimes

The operational lifetimes for all of Laos’ irrigated and rainfed crops were sourced from the aforementioned guidance document put together by Climate Compatible Growth [14].

#### 4.2.4 Output activity ratios

Table 39 outlines the sources used to calculate the output activity ratios for each of Laos’ land technologies.

**Table 39:** List of sources for the output activity ratios for land technologies.

Technology	Sources
All irrigated crops	[20,21]
All rainfed crops	[20,21]
All crop imports	N/A (left at default model value)
Forests	[24]
Built-up areas	[24]
Water bodies	[24]
Other land	[24]

As each of Laos’ crops is represented by two technologies (rainfed and irrigated) in the CLEWs model, it was necessary to calculate the ratio of productivity for each crop variation. The first step to do this was to calculate the area of irrigated land in Laos. This was determined by multiplying the ‘ % of cultivated area equipped for irrigation’ value provided in the AQUASTAT database [20] to Laos’ total land area size. The rainfed land was equivalent to this value subtracted from the total area harvested.

Eq. (5) shows how the output activity ratio, also referred to as the crop yield, was then calculated for rainfed crops. Eq. (6) shows how the ratio was calculated for irrigated crops. The ratio between irrigated and rainfed yields was also taken from the AQUASTAT database.

(5) $$Rainfed\;yield=\frac{Production\;quantity}{\left(Irrigated\;land*Ratio\;between\;irrigated\;and\;rainfed\;yields\right)+Rainfed\;land}$$

(6) Irrigatedyield=Rainfedyield×Ratiobetweenirrigatedandrainfedyields

#### 4.2.5 Residual capacities

The residual capacities of Laos’ crops were calculated by applying a linear reduction over 15 years to the respective crop’s 2020 area harvested value. After 15 years, which is equivalent to the operational lifetime of each crop, the residual capacity value equals 0.

Eq. (7) was used to calculate the area harvested for the irrigated variation of a crop. The irrigation potential was sourced from the AQUASTAT database [20] and the total area harvested for each crop was sourced from the Food and Agriculture Organisation’s (FAO) FAOSTAT dataset [12]. Eq. (8) was used to calculate the area harvested for the rainfed crop variation.

(7) Irrigatedareaharvested=Totalareaharvested*Irrigationpotential

(8) Rainfedareaharvested=Totalareaharvested*(100−Irrigationpotential)

### 4.3 Water

#### 4.3.1 Demands

Eq. (9) shows how the accumulated annual demand for Laos’ public water was calculated. The FAO AQUASTAT dataset [11] was used to source Laos’ historic water demand. Demand is projected to increase with the country’s GDP growth rate, based on the assumption that economic growth is often associated with increased urbanisation and industrial activity, which typically increases water use. This assumption is supported by observations made by Vanham et al. [25], that found a strong linear relationship between GDP and a country’s total water footprint. Readers should note that projecting accumulated annual demand for public water using this equation is limited because it assumes a linear relationship between GDP and water demand, and does not consider potential improvements in water use efficiency or behavioural changes.

(9) $$Annual\;water\;demand={\left(Current\;consumption\;\times GDP\;growth\;rate\;\%\right)}^{Years}$$

#### 4.3.2 Input activity ratios

As shown in Table 40, the input activity ratio for thermal cooling water in Laos’ fuel-based energy generation technologies was sourced from Climate Compatible Growth [24].

**Table 40:** List of sources for water-specific input activity ratios.

Technology	Sources
Thermal cooling water for Gas Plant (CCGT)	[24]
Thermal cooling water for Gas Plant (SCGT)	[24]
Thermal cooling water for Coal power plant	[24]
Precipitation water for all land technologies	[18,20]
Irrigation water for irrigated crop technologies	[20]

The input activity ratio for precipitation water was sourced using data on Laos’ long-term average annual precipitation in volume (10^9^m^3^) from the AQUASTAT database [20], and using Eq. (10) Total country land area is measured in 10^3^km^2^.

(10) $$\text{Precipitation}\;input\;activity\;ratio=\;\frac{Long\;term\;average\;annual\;precipitation}{Total\;country\;land\;area}$$

The input activity ratio for irrigation water was also calculated using data from the AQUASTAT database. As shown in Eq. (11), the irrigation water withdrawal value (10^9^m^3^) from the AQUASTAT database is divided by the total area equipped for full control irrigation (10^3^km^2^) to get the input activity ratio.

(11) $$\text{Irrigation}\;water\;input\;activity\;ratio=\;\frac{Irrigation\;water\;withdrawal}{Total\;area\;equipped\;for\;full\;control\;irrigation}$$

#### 4.3.3 Output activity ratios

The output activity ratios for water lost to evapotranspiration, groundwater recharge and surface water run-off in Laos’ land and crop technologies were taken from global averages compiled by Climate Compatible Growth, as no country-specific data was available. For rice, cassava, sugarcane, and maize, crop-specific output activity ratios were available. For the remaining crops, averages provided by Climate Compatible Growth were used [14].

## Limitations

Freely available and nationally endorsed data on Laos’ CLEW systems published within the last five years were limited; in particular, it was not possible to identify national data on crop production costs or on the water use and outputs of key crops. In these cases, international averages and estimates were used as proxy data. Future users should therefore consider this dataset a starting point for further refinement in building their own Laos CLEWs model, and it is suggested that national data replace international averages where possible. It is also recommended that the energy sector data be cross-validated with the most recently published datasets to improve accuracy, particularly regarding Laos’ existing power generation technologies. Finally, future users should consider the potential influence of long-term socio-economic drivers, like projected population growth, on techno-economic data inputs that have been assumed to remain constant, such as fixed costs.

## Ethics Statement

The authors of this article have read and follow the ethical requirements for publication in Data in Brief. The research work required for this article does not involve human subjects, animal experiments, or any data collected from social media platforms.

## CRediT Author Statement

**A. Flint Smith:** Methodology, Data curation, Writing, Original draft preparation. **F. A. Plazas-Niño:** Supervision, Writing – review and editing. **K. Alexander:** Supervision, Writing – review and editing. **M. Howells:** Supervision, Conceptualisation.

## Data Availability

ZenodoTechno-economic climate, land, energy and water systems dataset for long-term decarbonisation modelling in Laos (Original data).
